# Indoor air quality in a training centre used for sports practice

**DOI:** 10.7717/peerj.15298

**Published:** 2023-05-01

**Authors:** Victoria Mazoteras-Pardo, Marta Elena Losa-Iglesias, Israel Casado-Hernández, César Calvo-Lobo, Ángel Morales-Ponce, Alfredo Medrano-Soriano, Sergio Coco-Villanueva, Ricardo Becerro-de-Bengoa-Vallejo

**Affiliations:** 1 Department of Nursing, Physiotherapy and Occupational Therapy, Faculty of Physiotherapy and Nursing of Toledo, Toledo, Spain; 2 Department of Nursing, Faculty of Health Sciences. Universidad Rey Juan Carlos de Madrid, Madrid, Spain; 3 Department of Nursing, Faculty of Nursing, Physiotherapy and Podiatry, Universidad Complutense de Madrid, Madrid, Spain

**Keywords:** Air pollution, Exercise, Fitness centres, Indoor air quality

## Abstract

**Background:**

One of the measures for controlling the coronavirus disease 2019 (COVID-19) pandemic was the mass closure of gyms. This measure leads us to determine the differences between indoor and outdoor air quality. That is why the objective of this study was to analyse the indoor air quality of a sports centre catering to small groups and rehabilitation.

**Methods:**

The study was conducted in a single training centre, where 26 measurements were taken in two spaces (indoors and outdoors). The air quality index, temperature, relative humidity, total volatile compounds, carbon monoxide, ozone, formaldehyde, carbon dioxide, and particulate matter were measured indoors and outdoors using the same protocol and equipment. These measurements were taken twice, once in the morning and once in the afternoon, with all measurements made at the same time, 10 am and 6 pm, respectively. Additionally, four determinations of each variable were collected during each shift, and the number of people who had trained in the room and the number of trainers were counted.

**Results:**

In the different variables analysed, the results show that CO_2_ and RH levels are higher indoors than outdoors in both measurement shifts. Temperatures are higher outside than inside and, in the evening, than in the morning. TVOC, AQI and PM show less variation, although they are higher outdoors in the morning. CO is highest indoors. HCHO levels are almost negligible and do not vary significantly, except for a slight increase in the afternoon outside. Ozone levels are not significant. All the variables showed practically perfect reliability in all the measurements, except for ozone measured outside in the morning. On the other hand, the variables exhibit variations between indoors and outdoors during the morning and afternoon, except for the three types of PM. Also, the data show that all the main variables measured inside the sports training centre are similar between morning and afternoon. However, outside, temperature, relative humidity and HCHO levels show significant differences between morning and afternoon while no differences are observed for the other variables.

**Conclusion:**

The indoor air quality of the training centre assessed was good and met current regulations; some of its components even exhibited better levels than fresh air. This article is the first to measure indoor air quality in a sports training centre catering to rehabilitation and small groups.

## Introduction

In December 2019, a new coronavirus (SARS-CoV-2) was identified in Wuhan, Hubei province of China, causing the first cases of the disease now known as coronavirus disease 2019 (COVID-19). Since then, this disease has spread worldwide at an unprecedented speed, leading the World Health Organization (WHO) to declare it a global pandemic on March 11, 2020 (WHO, 2020; Phua et al., 2020).

Clinical experience indicates that COVID-19 is highly heterogeneous, with a range of symptoms from asymptomatic and mild to severe and causing death (Chen et al., 2021; Yüce, Filiztekin & Özkaya, 2021). From an individual perspective, restrictions on outdoor exercise due to geographical limitations and the need to stay indoors have reduced physical activity and increased sedentary behaviour (Colley, Bushnik & Langlois, 2020; Phillipou et al., 2020; Yeo, 2020).

It is well-known that physical exercise reduces cardiovascular risk factors and improves the immune system (Chowdhury et al., 2020). A weak immune system is vital in increasing vulnerability to severe COVID-19 consequences in older adults (Yeo, 2020). For these reasons, along with the importance of personal hygiene and social distancing, maintaining a healthy lifestyle is crucial to reducing the risk of COVID-19 infection, thereby promoting physical activity due to its indisputable physical and mental health benefits (Zhang et al., 2020; Nyenhuis et al., 2020; Yeo, 2020).

Despite the above, it may be challenging to reconcile these claims with the mass closure of gyms and training centres as an additional measure during the pandemic, coupled with the recommendation to avoid enclosed spaces (Colley, Bushnik & Langlois, 2020; Yeo, 2020). Furthermore, inspecting indoor training spaces revealed that it is unknown whether a conventional gymnasium has the same indoor air conditions and, in turn, the same likelihood of contagion as a small group training and rehabilitation centre (Andrade & Dominski, 2018).

In this regard, it is important to assess the air quality of these indoor spaces, as it plays a significant role in people’s health status. However, the literature has observed that air quality is more extensively analysed in other facilities, such as hospitals (El-Sharkawy & Noweir, 2014; Baurès et al., 2018). To evaluate indoor air quality, biological pollutants (moisture and mould), chemical pollutants, and indoor combustion pollutants must be taken into account (WHO, 2022). Air quality indices include temperature and relative humidity (RH), carbon dioxide (CO_2_) levels, total volatile compounds (TVOC) (Nordstrom, Norback & Akselsson, 1994), particulate matter (PM) (Buemi et al., 2000; Slezakova, Morais & do Pereira, 2014), bacteria (Buemi et al., 2000; Li & Hou, 2003; Wan, Lu & Tsai, 2004), fungi (Li & Hou, 2003; Afshari et al., 2013; Méheust, Le Cann & Gangneux, 2013), viruses (Tsai et al., 2006), and toxic metals (Slezakova, Morais & do Pereira, 2014). Temperature and relative humidity have been previously used to reflect the efficiency of hospital ventilation systems (El-Sharkawy & Noweir, 2014). On the other hand, CO_2_ concentration has been used as the most critical index to assess indoor air quality, correlating with the number of people in the space and PM concentrations (Scheff et al., 2000; Li, Lee & Chan, 2001) and has been significantly associated with airborne bacterial counts (Liu et al., 2000). In general, many authors identify temperature and RH as determinants when measuring air quality, along with other variables that determine indoor air quality in vulnerable environments: carbon monoxide (CO), CO_2_, ozone (O_3_), particles of different diameters (PM 2.5; PM 10), sulfur dioxide (SO_2_), and total volatile organic compounds.

Formaldehyde (HCHO), a volatile compound known as methanal, is used in the production of cleaning products and manufacturing materials and is naturally present in the environment (Nordstrom, Norback & Akselsson, 1994; El-Sharkawy & Noweir, 2014; Baurès et al., 2018).

Despite its importance, we have not found any recent studies in the scientific literature that measure air quality in sports training centres in spite of similar research have been done (Salonen et al., 2008; Alves et al., 2014; Szulc et al., 2023). Therefore, we aimed to innovate in terms of the objective and design of our study by analysing the air quality of an indoor sports training centre catering to small groups, considering its determining parameters at different times of the day and comparing them to the outdoor air quality.

## Materials and Methods

### Experimental site

The study was conducted in an indoor training centre equipped with top-brand materials, with COVID-free protocols in place. The centre was divided into two rooms with six independent training stations, each equipped with its own materials for personal use, which were disinfected after each training session. The two main rooms measure 200 square metres and 100 square metres. Each working area is approximately 25–30 square metres. Additionally, the centre had an evaluation room, changing rooms, air conditioning, and air recycling.

### Study design

We performed an observational study according to the STrengthening the Reporting of OBservational studies in Epidemiology (STROBE) criteria (Vandenbroucke et al., 2014).

### Ethical information

This study complied with the ethical principles of the Declaration of Helsinki (World Medical Association, 2013), including amendments from 2000 to 2013. The approval of an ethics committee was not required.

### Parameters

The main research variables collected in the study were the following: air quality index, temperature, RH, TVOC, HCHO, CO, CO_2_, PM 1.0, PM 2.5, PM 10, and O_3_.

### Devices

#### Air quality detector new JSM-131 CO

This natural air flow detector meets CE, RoHS, and FCC compliance standards. The error range is between 3% and 30%. It measures carbon monoxide (CO), carbon dioxide (CO_2_), HCHO, and total volatile organic compounds (TVOC) and indicates the air quality index (AQI). The error range of this device is approximately 3–30% (Saini, Dutta & Marques, 2021).

#### KKmoon® meter IHG7057623542157JX

This device detects PM less than 2.5 μg/m^3^ (PM 2.5), less than 1.0 μg/m^3^ (PM 1.0), and less than 10 μg/m^3^ (PM 10). The measurement range is 0–2,048 μg/m^3^, with an accuracy of 1 μg/m^3^ (Paterakis et al., 2022).

#### Carbon dioxide detector SR-510

The carbon dioxide detector SR-510 (AMSTAT, Tinton Falls, NJ, USA) uses the absorption principle of infrared light sources to detect CO_2_ gas in the onsite environment. This device also records temperature and relative humidity (RH) and is auto-calibrated. The data is displayed on an LED screen, and the device has an accuracy of ±40 ppm ± rdg 10%. For CO_2_, it has a detection range of 0–9,999 ppm and a resolution of 1 ppm. Temperature is measured in degrees Celsius (°C), with a measurement range of −20 °C to 60 °C (−4 °F to 140 °F), an accuracy of ±1 °C (±2 °F), and a resolution of 0.01 °C/°F. The RH range is 0–100% RH, and the humidity measurement precision is ±2% RG (20–80% RH), with a humidity resolution of 0.01% RH (Dervieux, Théron & Uhring, 2022).

#### Ozone meter

This multifunctional ozone meter detects O_3_ in parts per million (ppm), temperature, and humidity. It has an electrochemistry ozone sensor with a 0–5 ppm test range.

### Sample size

The sample size was calculated using G*Power 3.1.9.4 software (University of Kiel, Kiel, Germany). Pearson’s test was used to correlate the air quality index in indoor gyms with the quality of the outside air, assuming a normal distribution with a two-tailed test, an alpha error of 0.05, a confidence level of 95%, and a desired statistical power of 80% (beta error = 20%). An effect size of 0.5 was used, and 26 measurements were taken at each location (indoors and outdoors).

### Study protocol

The following variables were measured inside the centre as well as outside: CO, CO_2_, HCHO, TVOC, AQI, PM 1.0, PM 2.5, PM 10, O_3_, temperature, and relative humidity using the abovementioned devices.

All devices were installed at 1.50 m, following the current monitoring guidelines in other environments (World Health Organization & Regional Office for Europe, 2010). The measurements were taken over 26 days during two shifts: morning and afternoon. All measurements were taken simultaneously, at 10 am and 6 pm. Four measurements were taken for each variable during each shift. Indoors, the number of people who trained in the room and the number of trainers were counted during each shift. Outside, the measurement point was on the street, in front of the training centre (Rda. de Alarcos, 38, 13002 Ciudad Real). There are no industries nearby, only blocks of flats and shops. Also, it is a street with car and pedestrian traffic.

In addition, the Ciudad Real Air Quality Index (AQI) (European Environment Agency, 2021), created in 2017 with the European Environment Agency, was also measured to assess air quality. This measurement considers the five key pollutants harmful to human health and the environment: PM 2.5, PM 10, O_3_, nitrogen dioxide (NO_2_), and sulfur dioxide (SO_2_). The AQI scale ranges from 0 to >500 and establishes six categories. Air quality is worse the higher the index: Good (0–50), Moderate (51–100), Unhealthy for Sensitive Groups (101–150), Unhealthy (151–200), Very Unhealthy (201–300), and Hazardous (>300). This index is provided by the European Environment Agency. This value and that of its five components can be consulted on the website, which has several reference points in the real city and provides you with the closest result to the location, the training centre.

### Data analysis

The data were analysed using the SPSS for Windows statistical package version 22.0 (SPSS, Inc., Chicago, IL, USA). A *P*-value of <0.05 was considered statistically significant with a confidence interval of 95%. The Shapiro-Wilk test was used for sample sizes less than 50 to determine whether the quantitative variables followed a normal distribution (Vetter, 2017). The mean and standard deviation (SD) were used to describe all quantitative variables with a confidence level of 95%, as well as the median, interquartile range, and range.

The intraclass correlation coefficient (ICC) was used to calculate the reliability of the variables included in the study, both in the morning and afternoon and indoors and outdoors. To interpret the ICC values, the convergence reference points proposed by Landis and Koch were used: 0.20 or less, slight; 0.21 to 0.40, fair; 0.41 to 0.60, moderate; 0.61 to 0.80, substantial; and 0.81 or more, almost perfect (Landis & Koch, 1977). We used the Portney and Watkins guide (Portney & Watkins, 2009), which indicates that clinical measurements with a coefficient of over 0.90 increase the probability of the reliability of the measures.

The coefficient of variation (CV) (Portney & Watkins, 2009) was measured using the formula CV% = (SD/mean) × 100, where SD represents the standard deviation and mean represents the average of the variable being studied. The CV indicates the degree of variability concerning the mean size, expressed as a percentage.

The standard error of measurement (SEM) was also calculated for each main parameter to estimate the amount of error in each measurement. This was calculated using the following formula (Hamilton & Stamey, 2007): $\rm {SEM = DS * \sqrt {(1 - ICC)}}$, where DS is the standard deviation of each variable and ICC is the intraclass correlation coefficient for each variable. A lower SEM value indicates a lower error in the measurement.

The minimum detectable change (MDC) was also calculated to determine the smallest change in the value of each scale that could be interpreted as a true change in the room’s air quality. MDC is defined as the size of change below which the change can be attributed to the variability of the measurement method. The MDC was calculated using a standardised mean (MDC 95%) (Jacobson & Truax, 1991) as follows: $\rm {MDC = 1.96 * SEM * \sqrt 2}$, where SEM is the standard measurement error for each variable. The statistical significance was considered for values of *P* < 0.05.

The main variables of the study were compared between indoor and outdoor environments in the morning and afternoon times using the non-parametric Mann-Whitney U test for independent samples (Hazra & Gogtay, 2016).

## Results

After performing the Shapiro-Wilk test, it was found that all outcome variables studied had a non-normal distribution (*P* < 0.05). Tables 1–4 present the descriptive statistics of the main variables measured using all the study equipment in different places (indoors and outdoors) and times (morning and afternoon). The different parameters show that CO_2_ and RH levels are higher indoors than outdoors in both measurement shifts. Among the two, the highest figures were observed indoors, with CO_2_ being the highest in the afternoon and humidity in the morning. The lowest figures were obtained outdoors in the afternoon. RH values are notable for their very different figures, as can be observed from their measurement ranges, with minimum and maximum figures being far apart in both places and times. Temperature is the opposite, with higher levels observed outside in the afternoon, with a maximum temperature of over 41 °C. TVOC, AQI, and PM show less variation, although they are higher outdoors in the morning.

**Table 1:** Descriptors of the variables analysed indoors at the Centre in the morning.

Variable	Mean ± SD	(95% CI)	Median (IR)	Min-Max
CO_2_	632.46 ± 106.31	[568.22–696.70]	608 (188.13)	497.75–843.75
Temperature	25.09 ± 0.42	[24.85–25.35]	25.09 (0.38)	24.40–26.20
%RH	40.92 ± 7.23	[36.55–45.30]	39.46 (0.27)	30.99–52.62
TVOC	0.57 ± 0.87	[0.05–1.10]	0.15 (0.93)	0–2.88
AQI	2.56 ± 0.94	[1.99–3.12]	2.00 (1.50)	1.50–4.25
CO	13.19 ± 18.43	[2.06–24.33]	4.50 (17.0)	2.0–58.0
HCHO	0.05 ± 0.06	[0.01–0.08]	0 (0.07)	0–0.19
PM 2.5	14.79 ± 7.14	[10.47–19.10]	12.75 (11.38)	4.75–30.75
PM 1.0	9.33 ± 3.96	[6.94–11.72]	8.50 (5.50)	2.75–16.0
PM 10	15.52 ± 7.50	[11.0–20.05]	13.25 (10.75)	5.50–33.0
O_3_	0 ± 0.01	[0–0.01]	0 (0.01)	0–0.02

**Note:**

SD, standard deviation; IR, interquartile range; CO_2_, carbon dioxide; RH%, relative humidity percentage; TVOC, total volatile organic compounds; AQI, air quality index; CO, carbon monoxide; HCHO, formaldehyde; PM 2.5, particulates of less than 2.5 ug/m^3^; PM 1.0, particulates of less than 1.0 ug/m^3^; PM 10, particulates of less than 10 ug/m^3^; O_3_, ozone.

**Table 2:** Descriptors of the variables analysed outdoors in the morning.

Variable	Mean ± SD	(95% CI)	Median (IR)	Min-Max
CO_2_	438.88 ± 13.82	[430.53–447.24]	439.50 (17.13)	414.75–467.75
Temperature	29.29 ± 2.76	[27.62–30.96]	29.49 (4.70)	25.0–33.32
%RH	30.84 ± 8.38	[25.77–35.90]	29.16 (11.94)	18.99–49.35
TVOC	2.53 ± 3.09	[0.67–4.41]	1.83 (1.66)	0.03–12.0
AQI	3.60 ± 1.20	[2.87–4.32]	4.00 (1.50)	2.0–6.0
CO	134.67 ± 181.27	[25.13–244.22]	98.25 (105.63)	10.50–683.0
HCHO	0.11 ± 0.09	[0.05–0.16]	0.08 (0.13)	0.01–0.33
PM 2.5	20.77 ± 12.41	[13.27–28.27]	17.50 (19.38)	3.50–44.0
PM 1.0	13.58 ± 8.46	[8.46–18.69]	10.75 (11.50)	2.0–28.75
PM 10	22.73 ± 15.41	[13.42–32.04]	16.50 (21.25)	3.75–53.0
O_3_	0.01 ± 0.01	[0.01–0.012]	0.01 (0.01)	0–0.02

**Note:**

SD, standard deviation; IR, interquartile range; CO_2_, carbon dioxide; RH%, relative humidity percentage; TVOC, total volatile organic compounds; AQI, air quality index; CO, carbon monoxide; HCHO, formaldehyde; PM 2.5, particulates of less than 2.5 ug/m^3^; PM 1.0, particulates of less than 1.0 ug/m^3^; PM 10, particulates of less than 10 ug/m^3^; O_3_, ozone.

**Table 3:** Descriptors of the variables analysed indoors at the Centre in the afternoon.

Variable	Mean ± SD	(95% CI)	Median (IR)	Min-Max
CO_2_	714.85 ± 148.83	[624.91–804.78]	670.5(261.75)	502.0–1,000.25
Temperature	25.94 ± 0.87	[25.41–26.46]	25.75 (1.44)	24.91–27.79
%RH	36.29 ± 4.85	[33.35–39.22]	38.65 (8.32)	27.02–41.47
TVOC	0.29 ± 0.55	[0.04–0.62]	0.15 (0.11)	0–2.10
AQI	2.11 ± 0.58	[1.76–2.47]	2.00 (0)	1.50–4.0
CO	13.36 ± 28.91	[4.10–30.83]	5.50 (2.25)	2.0–109.0
HCHO	0.02 ± 0.04	[0–0.04]	0.01 (0.01)	0–0.14
PM 2.5	9.01 ± 3.76	[6.75–11.29]	9 (6.0)	3.50–15.50
PM 1.0	6.10 ± 2.62	[4.51–7.68]	6.25 (4.88)	2.0–10.25
PM 10	9.48 ± 3.96	[7.09–11.87]	10.25 (6.13)	3.50–15.75
O_3_	0 ± 0	[0–0.01]	0 (0.01)	0–0.02

**Note:**

SD, standard deviation; IR, interquartile range; CO_2_, carbon dioxide; RH%, relative humidity percentage; TVOC, total volatile organic compounds; AQI, air quality index; CO, carbon monoxide; HCHO, formaldehyde; PM 2.5, particulates of less than 2.5 ug/m^3^; PM 1.0, particulates of less than 1.0 ug/m^3^; PM 10, particulates of less than 10 ug/m^3^; O_3_, ozone.

**Table 4:** Descriptors of the variables analysed outdoors in the afternoon.

Variable	Mean ± SD	(95% CI)	Median (IR)	Min-Max
CO_2_	432.62 ± 9.73	[426.74–438.49]	430.75 (16.50)	421.0–454.0
Temperature	35.94 ± 3.82	[33.64–38.25]	35.26 (3.83)	30.29–41.12
%RH	19.86 ± 5.98	[16.25–23.47]	19.75 (9.09)	9.62–31.11
TVOC	1.40 ± 1.27	[0.64–2.17]	1.35 (1.61)	0–4.68
AQI	2.96 ± 0.97	[2.37–3.55]	2.75 (1.75)	2.0–5.0
CO	79.08 ± 83.31	[28.73–129.42]	47.25 (105.13)	2.0–312.5
HCHO	11.20 ± 40.05	[13.0–35.40]	0.08 (0.12)	0–144.5
PM 2.5	12.04 ± 9.04	[6.58–17.50]	8.50 (11.63)	3.50–33.25
PM 1.0	7.87 ± 5.51	[4.54–11.19]	7.0 (7.38)	2.25–20.50
PM 10	12.77 ± 9.87	[6.80–18.74]	11.50 (10.0)	3.75–38.75
O_3_	0.04 ± 0.05	[0.01–0.06]	0.02 (0.04)	0–0.16

**Note:**

SD, standard deviation; IR, interquartile range; CO_2_, carbon dioxide; RH%, relative humidity percentage; TVOC, total volatile organic compounds; AQI, air quality index; CO, carbon monoxide; HCHO, formaldehyde; PM 2.5, particulates of less than 2.5 ug/m^3^; PM 1.0, particulates of less than 1.0 ug/m^3^; PM 10, particulates of less than 10 ug/m^3^; O_3_, ozone.

The results show a clear difference in CO levels indoors and outdoors, with much higher levels inside the gym. Outside the gym, there is more variation in CO levels between morning and afternoon, while the figures are relatively consistent inside. HCHO levels are almost negligible and do not vary significantly except for a slight increase in the afternoon outside. Ozone levels are not significant.

Tables 5 and 6 present the descriptors of the outdoor air in Ciudad Real, based on the AQI created by the European Environmental Agency and its five pollutants in the morning and afternoon. AQI and O_3_ are lower in the morning than in the afternoon, while NO_2_ and SO_2_ are higher in the morning, with smaller differences. PM levels are similar in both shifts. Tables 7–10 display the analysis of the reliability of the equipment used in the four measurements of each variable, indoors and outdoors, at the training centre in the morning and afternoon. All the variables showed practically perfect reliability in all the measurements, except for ozone measured outside in the morning, which had significant reliability (ICC = 0.663). It was observed that CO_2_ and RH had a maximum level of correlation between the measurements taken indoors and outdoors in the morning (ICC = 1), indicating that the values remain constant over the four measurements of each shift and are, therefore, accurate.

**Table 5:** Descriptors of the variables studied outdoors (European Environment Agency) in the morning.

Variable	Mean ± SD	(95% CI)	Median (IR)	Min-Max
AQI	37.84 ± 18.47	[26.69–49.0]	34.0 (18.50)	20.0–92.0
O_3_	32.23 ± 7.93	[27.44–37.02]	32.20 (9.0)	20.40–48.60
NO_2_	9.18 ± 3.72	[6.93–11.43]	9.20 (3.0)	4.70–19.40
SO_2_	4.34 ± 13.87	[4.04–12.72]	0.60 (0.40)	0.10–50.50
PM 2.5	20.24 ± 7.77	[15.55–24.94]	18.40 (7.75)	10.70–39.20
PM 10	33.86 ± 18.94	[22.42–45.31]	28.70 (11.15)	20.40–92.10

**Note:**

SD, standing deviation; IR, interquartile range; AQI, air quality index; O_3_, ozone; PM2.5, particulates of less than 2.5 ug/m^3^; PM 10, particulates of less than 10 ug/m^3^; NO_2_, nitrogen dioxide; SO_2_, sulphur dioxide.

**Table 6:** Descriptors of the variables studied outdoors (European Environment Agency) in the afternoon.

Variable	Mean ± SD	(95% CI)	Median (IR)	Min-Max
AQI	50.31 ± 17.81	[39.55–61.06]	46.0 (18.50)	25.0–88.0
O_3_	50.76 ± 17.84	[39.98–61.54]	46.80 (18.05)	25.50–88.50
NO_2_	5.96 ± 1.32	[5.17–6.76]	6.0 (1.40)	3.50–8.40
SO_2_	0.48 ± 0.17	[0.38–0.59]	0.50 (0.15)	0.10–0.70
PM 2.5	20.25 ± 9.76	[14.35–26.14]	20.20 (11.05)	8.20–45.20
PM 10	31.39 ± 16.26	[21.57–41.22]	29.80 (15.80)	11.20–77.10

**Note:**

SD, standing deviation; IR, interquartile range; AQI, air quality index; O_3_, ozone; PM 2.5, particulates of less than 2.5 ug/m^3^; PM 10, particulates of less than 10 ug/m^3^; NO_2_, nitrogen dioxide; SO_2_, sulphur dioxide.

**Table 7:** Reliability of the variables analysed indoors at the Centre in the morning.

Variable	ICC (95% CI)	CV	SEM	MDC
CO_2_	1 [0.999–1.000]	0.168	0.000	0.0000
Temperature	0.997 [0.993–0.999]	0.017	0.023	0.064
%RH	0.999 [0.997–1.000]	0.177	0.229	0.634
TVOC	0.997 [0.994–0.999]	1.526	0.048	0.132
AQI	0.934 [0.845–0.977]	0.328	0.216	0.598
CO	0.992 [0.981–0.997]	1.397	1.648	4.569
HCHO	0.996 [0.991–0.999]	1.200	0.004	0.011
PM 2.5	0.981 [0.956–0.994]	0.483	0.984	2.728
PM 1.0	0.985 [0.966–0.995]	0.424	0.485	1.344
PM 10	0.978 [0.949–0.992]	0.483	1.112	3.084
O_3_	0.908 [0.785–0.968]	0	0.003	0.008

**Note:**

CI, confidence interval; ICC, intraclass correlation coefficients; SEM, standard error measurement; MDC, minimum detectable change; CV, coefficient of variation; CO_2_, carbon dioxide; RH%, relative humidity percentage; TVOC, total volatile organic compounds; AQI, air quality index; CO, carbon monoxide; HCHO, formaldehyde; PM 2.5 particulates less than 2.5 ug/m^3^; PM 1.0, particulates less than 1.0 ug/m^3^; PM 10, particulates less than 10 ug/m^3^; O_3_, ozone.

**Table 8:** Reliability of the variables studied outdoors in the morning.

VARIABLE	ICC (95% CI)	CV	SEM	MDC
CO_2_	0.964 [0.915–0.988]	0.032	2.622	7.268
Temperature	0.998 [0.995–0.999]	0.094	0.123	0.342
%RH	1.000 [0.999–1.000]	0.272	0.000	0.000
TVOC	0.997 [0.994–0.999]	1.221	0.169	0.469
AQI	0.972 [0.936–0.991]	0.333	0.201	0.557
CO	0.995 [0.988–0.998]	1.346	12.818	35.529
HCHO	0.954 [0.893–0.984]	0.818	0.019	0.054
PM 2.5	0.989 [0.974–0.996]	0.598	1.302	3.608
PM 1.0	0.989 [0.975–0.996]	0.623	0.887	2.459
PM 10	0.989 [0.975–0.996]	0.678	1.616	4.480
O_3_	0.663 [0.214–0.885]	1.000	0.006	0.016

**Note:**

CI, confidence interval; ICC, intraclass correlation coefficients; SEM, standard error measurement; MDC, minimum detectable change; CV, coefficient of variation; CO_2_, carbon dioxide; RH%, relative humidity percentage; TVOC, total volatile organic compounds; AQI, air quality index; CO, carbon monoxide; HCHO, formaldehyde; PM 2.5 particulates less than 2.5 ug/m^3^; PM 1.0, particulates less than 1.0 ug/m^3^; PM 10, particulates less than 10 ug/m^3^; O_3_, ozone.

**Table 9:** Reliability of the variables analysed indoors at the Centre in the afternoon.

VARIABLE	ICC (95% CI)	CV	SEM	MDC
CO_2_	0.999 [0.999–1.000]	0.208	4.706	13.046
Temperature	0.971 [0.933–0.990]	0.034	0.148	0.411
%RH	0.971 [0.933–0.990]	0.134	0.826	2.289
TVOC	0.997 [0.992–0.999]	1.897	0.0301	0.084
AQI	0.943 [0.868–0.981]	0.275	0.139	0.384
CO	0.997 [0.992–0.999]	2.164	1.584	4.389
HCHO	0.996 [0.992–0.999]	2.000	0.003	0.007
PM 2.5	0.974 [0.939–0.991]	0.417	0.606	1.681
PM 1.0	0.984 [0.962–0.994]	0.429	0.331	0.919
PM 10	0.975 [0.941–0.991]	0.418	0.626	1.736
**O**_**3**_	0.902 [0.772–0.966]	0	0.000	0.000

**Note:**

CI, confidence interval; ICC, intraclass correlation coefficients; SEM, standard error measurement; MDC, minimum detectable change; CV, coefficient of variation; CO_2_, carbon dioxide; RH%, relative humidity percentage; TVOC, total volatile organic compounds; AQI, air quality index; CO, carbon monoxide; HCHO, formaldehyde; PM 2.5 particulates less than 2.5 ug/m^3^; PM 1.0, particulates less than 1.0 ug/m^3^; PM 10, particulates less than 10 ug/m^3^; O_3_, ozone.

**Table 10:** Reliability of the variables analysed outdoors in the afternoon.

Variable	ICC (95% CI)	CV	SEM	MDC
CO_2_	0.965 [0.919–0.988]	0.0225	1.820	5.046
Temperature	0.995 [0.989–0.998]	0.1063	0.270	0.749
%RH	0.999 [0.998–1.000]	0.3011	0.189	0.524
TVOC	0.973 [0.937–0.991]	0.9071	0.209	0.578
AQI	0.940 [0.859–0.979]	0.3277	0.238	0.659
CO	0.977 [0.947–0.992]	1.0535	12.635	35.021
HCHO	0.943 [0.867–0.981]	3.5759	9.562	26.504
PM 2.5	0.926 [0.828–0.975]	0.7508	2.459	6.816
PM 1.0	0.919 [0.811–0.972]	0.7001	1.568	4.347
PM 10	0.911 [0.793–0.970]	0.7729	2.945	8.162
O_3_	0.926 [0.828–0.975]	1.2500	0.014	0.038

**Note:**

CI, confidence interval; ICC, intraclass correlation coefficients; SEM, standard error measurement; MDC, minimum detectable change; CV, coefficient of variation; CO_2_, carbon dioxide; RH%, relative humidity percentage; TVOC, total volatile organic compounds; AQI, air quality index; CO, carbon monoxide; HCHO, formaldehyde; PM 2.5 particulates less than 2.5 ug/m^3^; PM 1.0, particulates less than 1.0 ug/m^3^; PM 10, particulates less than 10 ug/m^3^; O_3_, ozone.

Tables 11 and 12 present the differences in the indoor and outdoor air quality at the training centre during morning and afternoon hours. Statistically significant differences were found in all variables between the two environments and times of day (*P* < 0.05), except for particulates (*P* > 0.05). In other words, the variables exhibit variations between indoors and outdoors during the morning and afternoon, except for the three types of PM.

**Table 11:** Comparison of the variables studied indoors and outdoors at the Centre in the morning.

Variable	MORNING, INDOOR Mean ± SD	MORNING, OUTDOOR Mean ± SD	*P* value
CO_2_	632.46 ± 106.31	438.88 ± 13.82	0.001
Temperature	25.09 ± 0.42	29.29 ± 2.76	0.002
%RH	40.92 ± 7.23	30.84 ± 8.38	0.002
TVOC	0.57 ± 0.87	2.53 ± 3.09	0.011
AQI	2.56 ± 0.94	3.60 ± 1.20	0.032
CO	13.19 ± 18.43	134.67 ± 181.27	0.001
HCHO	0.05 ± 0.06	0.11 ± 0.09	0.016
PM 2.5	14.79 ± 7.14	20.77 ± 12.41	0.136
PM 1.0	9.33 ± 3.96	13.58 ± 8.46	0.278
PM 10	15.52 ± 7.50	22.73 ± 15.41	0.239
O_3_	0 ± 0.01	0.01 ± 0.01	0.033

**Note:**

SD, standard deviation; CO_2_, carbon dioxide; RH%, percentage of relative humidity; TVOC, total volatile compounds; AQI, air quality index; CO, carbon monoxide; HCHO, formaldehyde; PM 2.5, particulates of less than 2.5 ug/m^3^; PM 1.0, particulates of less than 1.0 ug/m^3^; PM 10, particulates of less than 10 ug/m^3^; O_3_, ozone. Statistically significant with a value of *P* < 0.05 and a confidence interval of 95%.

**Table 12:** Comparison of the variables studied indoors and outdoors at the Centre in the afternoon.

Variable	INDOORS, AFTERNOON Mean ± SD	OUTDOORS, AFTERNOON Mean ± SD	*P* value
CO_2_	714.85 ± 148.83	432.62 ± 9.73	0.001
Temperature	25.94 ± 0.87	35.94 ± 3.82	0.001
%RH	36.29 ± 4.85	19.86 ± 5.98	0.001
TVOC	0.29 ± 0.55	1.40 ± 1.27	0.004
AQI	2.11 ± 0.58	2.96 ± 0.97	0.046
CO	13.36 ± 28.91	79.08 ± 83.31	0.005
HCHO	0.02 ± 0.04	11.20 ± 40.05	0.009
PM 2.5	9.01 ± 3.76	12.04 ± 9.04	0.328
PM 1.0	6.10 ± 2.62	7.87 ± 5.51	0.309
PM 10	9.48 ± 3.96	12.77 ± 9.87	0.401
O_3_	0 ± 0	0.04 ± 0.05	0.003

**Note:**

SD, standard deviation; CO_2_, carbon dioxide; RH%, relative humidity percentage; TVOC, total volatile organic compounds; AQI, air quality index; CO, carbon monoxide; HCHO, formaldehyde; PM 2.5, particulates of less than 2.5 ug/m^3^; PM 1.0, particulates of less than 1.0 ug/m^3^; PM 10, particulates of less than 10 ug/m^3^; O_3_, ozone. Statistical significance for a value of *P* < 0.05, with a confidence interval of 95%.

Table 13 shows the differences in air quality between morning and afternoon measurements taken indoors at the training centre, while Table 14 shows the differences in air quality between morning and afternoon measurements taken outdoors. The data in the two tables shows that all main variables measured indoors at the sports training centre are similar between morning and afternoon (*P* > 0.05). However, outdoors, the temperature, relative humidity, and HCHO levels show significant differences between morning and afternoon (*P* < 0.05), while no significant differences are observed in the other variables. Notably, CO_2_ shows a *P*-value of 1 in both comparisons.

**Table 13:** Comparison of the variables analysed in the morning and afternoon indoors at the Centre.

Variable	INDOORS, MORNING Mean ± SD	INDOORS, AFTERNOON Mean ± SD	*P* value
CO_2_	632.46 ± 106.31	714.85 ± 148.83	1.000
Temperature	25.09 ± 0.42	25.94 ± 0.87	0.053
%RH	40.92 ± 7.23	36.29 ± 4.85	0.074
TVOC	0.57 ± 0.87	0.29 ± 0.55	1.000
AQI	2.56 ± 0.94	2.11 ± 0.58	1.000
CO	13.19 ± 18.43	13.36 ± 28.91	1.000
HCHO	0.05 ± 0.06	0.02 ± 0.04	1.000
PM 2.5	14.79 ± 7.14	9.01 ± 3.76	0.060
PM 1.0	9.33 ± 3.96	6.10 ± 2.62	0.055
PM 10	15.52 ± 7.50	9.48 ± 3.96	0.063
O_3_	0 ± 0.01	0 ± 0	1.000

**Note:**

SD, standard deviation; CO_2_, carbon dioxide; RH%, relative humidity percentage; TVOC, total volatile organic compounds; AQI, air quality index; CO, carbon monoxide; HCHO, formaldehyde; PM 2.5, particulates of less than 2.5 ug/m^3^; PM 1.0, particulates of less than 1.0 ug/m^3^; PM 10, particulates of less than 10 ug/m^3^; O_3_, ozone. Statistical significance for a value of *P* < 0.05, with a confidence interval of 95%.

**Table 14:** Comparison of the variables analysed in the morning and afternoon outdoors.

Variable	OUTDOORS, MORNING Mean ± SD	OUTDOORS, AFTERNOON Mean ± SD	*P* value
CO_2_	438.88 ± 13.82	432.62 ± 9.73	1.000
Temperature	29.29 ± 2.76	35.94 ± 3.82	0.001
%RH	30.84 ± 8.38	19.86 ± 5.98	0.011
TVOC	2.53 ± 3.09	1.40 ± 1.27	1.000
AQI	3.60 ± 1.20	2.96 ± 0.97	0.958
CO	134.67 ± 181.27	79.08 ± 83.31	1.000
HCHO	0.11 ± 0.09	11.20 ± 40.05	0.043
PM 2.5	20.77 ± 12.41	12.04 ± 9.04	0.276
PM 1.0	13.58 ± 8.46	7.87 ± 5.51	0.289
PM 10	22.73 ± 15.41	12.77 ± 9.87	0.415
O_3_	0.01 ± 0.01	0.04 ± 0.05	0.167

**Note:**

SD, standard deviation; CO_2_, carbon dioxide; RH%, relative humidity percentage; TVOC, total volatile compounds; AQI, air quality index; CO, carbon monoxide; HCHO, formaldehyde; PM 2.5, particulates of less than 2.5 ug/m^3^; PM 1.0, particulates of less than 1.0 ug/m^3^; PM 10, particulates of less than 10 ug/m^3^; O_3_, ozone. Statistically significant for a value of *P* < 0.05, with a confidence interval of 95%.

## Discussion

Air quality is attracting increasing research attention because of its impact on health (Morawska et al., 2013; El-Sharkawy & Noweir, 2014; Baurès et al., 2018; Mannan & Al-Ghamdi, 2021). However, to our knowledge, the study has addressed air quality at indoor sports training centres (Alves et al., 2014; Szulc et al., 2023). This novel research assesses indoor air quality at a sports training centre for small groups and rehabilitation, comparing these readings with the outdoor environment. We identified previous research about the same field but performed in different places not used for sports and exercise (Li et al., 2021; Zhang et al., 2021).

It is important to assess the air quality of these centres occupied by persons to promote their health. Moreover, despite the great benefits provided by physical exercise for health (Zhang et al., 2020; Nyenhuis et al., 2020; Chowdhury et al., 2020; Halle et al., 2021), these centres were closed during the COVID-19 pandemic (Colley, Bushnik & Langlois, 2020; Yeo, 2020).

We found that, except for particles in suspension, there are large significant differences in all contaminants when comparing the two studied spaces. In both the morning and afternoon, the level of contaminants in the air outdoors was higher than the levels indoors, except for CO_2_.

In particular, there is an even bigger difference in CO_2_, as outdoors, it easily exceeds the levels recommended by the WHO and established by Royal Decree 1073/2002 on the assessment and management of environmental air quality, of 10 mg/m^3^ or 9 ppm (Agencia Estatal Boletín Oficial del Estado, 2004; Krzyzanowski & Cohen, 2008; WHO, 2010). This may be due to traffic, greater human activity, and other factors such as tobacco (Dickey, 2000). Some studies point to traffic as the main source of air pollution in urban zones, resulting in adverse effects on human health (El-Sharkawy & Noweir, 2014). Moreover, highly populated indoor areas must have effective ventilation systems, whose use has lately been increased to prevent the propagation of SARS-CoV-2 (Rodríguez et al., 2021; Berry et al., 2022).

All the TVOC and HCHO measurements were within acceptable limits under the UNE 171330-2: 2009 Standard on Inspection Procedures for environmental air quality indoors, which are TVOC < 200 µg/m^3^ and HCHO < 0.12 mg/m^3^ (AENOR, 2009). These components are related to disinfection and cleaning products (El-Sharkawy & Noweir, 2014), which have become extremely common during the COVID-19 pandemic; in the training centre under examination, all the materials are disinfected after use.

Concerning AQI and ozone, the measurements were low in all cases and were, therefore, within the range of good air quality, from 0 to 50 (30), although it can be seen that the air quality was better in the morning than the afternoon, and even reached moderate levels at that time (European Environment Agency, 2021). The AQI may be based on different air quality parameters and is used to improve the communication of health risks for the general population, although it may overestimate the overall effect of multiple pollutants (Xu et al., 2020).

In terms of particulates, the literature suggests that the highest levels of particulate emissions, PM 10, increase with greater human activity (El-Sharkawy & Noweir, 2014; Licina et al., 2016) and when more vigorous activities such as walking or cleaning (Ferro, Kopperud & Hildemann, 2004; Bhangar et al., 2016) are carried out, likely because of a combination of loss of skin, hair and clothing particles and mobilisation of particles from the ground and other contact surfaces (Braniš, Řezáčová & Domasová, 2005). Despite this, the concentrations of PM 10, PM 2.5, and PM 1 included in our study were low and within the acceptable range (AENOR, 2009; Agencia Estatal Boletín Oficial del Estado, 2004), both outdoors and indoors. This suggests efficient particulate filtration, relatively low occupancy, and the application of adequate hygiene protocols. The levels of particulate material were lower in the centre studied than the concentrations found in other indoor environments, such as hospitals, homes for the elderly, and schools (Morawska et al., 2013; El-Sharkawy & Noweir, 2014; Baurès et al., 2018; Hwang & Park, 2020; Lee, Lee & Kim, 2020; Mannan & Al-Ghamdi, 2021).

CO_2_ is the only variable whose figures are higher indoors, but in both morning and afternoon, they are still within recommended levels and the typical range of well-ventilated indoor environments (<750 ppm) (AENOR, 2009; Seppanen, Fisk & Mendell, 1999; Hwang & Park, 2020). The factors that influence the variability of CO_2_ levels include variable rates of air recirculation and unequal generation due to human metabolism concerning occupation and activities (Licina et al., 2016; Perez et al., 2018; Palacios Temprano et al., 2020).

Other determinants of air quality are relative humidity and temperature. Although the current coronavirus pandemic has further highlighted the importance of the correct operation of improved air quality systems, there is still a lack of information and specific guidelines on the handling of temperature and the percentage of humidity concerning the air for controlling SARS-CoV-2 (AENOR, 2009; Seppanen, Fisk & Mendell, 1999; Alhazzani et al., 2020; Elsaid & Ahmed, 2021).

The evidence recommends maintaining the level of humidity at between 40% and 60% for the whole year (AENOR, 2009; Seppanen, Fisk & Mendell, 1999; El-Sharkawy & Noweir, 2014; Perez et al., 2018; Palacios Temprano et al., 2020). In our research, this parameter appears higher indoors, given that, as shown in the results, the outdoor environment at the measurement site was too dry in August. As a result, the indoor levels were better than the outdoor levels.

Traditionally, it was considered that the best comfort temperature indoors was 22–25 °C. However, since 2004, the adaptive thermal comfort model, which defines the comfort temperature indoors based on user exposure to the outdoor temperature, increased this range to 21–29 °C (Jay et al., 2021). In addition, it should be recalled that body temperature increases when engaged in sports activity, so air conditioning systems must be in place to maintain the environmental temperature as low as possible within these thermal comfort margins. This maintenance of adequate environmental temperature systems, together with an effective airflow to help dissipate body heat through convection and evaporation of sweat, will allow users to complete their physical training without the premature fatigue caused by high thermal stress (Nielsen et al., 1993). Considering the above temperature values indoors for optimal exercise, our centre complies with these standards.

Moreover, it has been shown that the different parameters composing the air must provide reliability (Andrade & Dominski, 2018; European Environment Agency, 2021; Mannan & Al-Ghamdi, 2021), in other words, remain constant for an extended time range. For this reason, our research has also valued the reliability of all the variables studied and found that all are very reliable in all measurements.

Finally, to conclude this section, it is worth highlighting the limitations and strenghts of our study. The results of this study cannot be extrapolated to other indoor training centres, such as conventional gyms, given that the facilities and conditions in which physical activity is carried out are different. Outdoor measurements are also not comparable with other cities and times of the year. Another limitation of the study is that the spread of diseases by air has not been analysed. It would be useful to analyse the air quality in different spaces where people engage in physical exercise and thus be able to compare them. Also, other objectives, such as the transmission of respiratory diseases, were considered. On the other hand, we present the main strengths of our study: the methodology is novel, since it examines the determinant variables of both outdoor and indoor air quality and compares them. In addition, reliability is determined as the study makes repeated measurements. The measurement site is also atypical. In fact, there are no previous studies published in gyms like the one addressed, nor in Ciudad Real.

## Conclusion

This study is the first to analyse the indoor air quality of a sports centre designed for small groups and rehabilitation. Based upon the results obtained and current regulations, the indoor air quality of this centre is adequate, considering the variables studied. Some of its components show even better values than those of fresh air. Therefore, the air quality in this centre is suitable for sports practice, although other factors not analysed, such as the transmission of diseases through the air, should also be considered.

## Supplemental Information

10.7717/peerj.15298/supp-1 Supplemental Information 1 Raw Data.

## Abbreviations

WHO: World Health Organization
COVID-19: Coronavirus disease 2019
CO_2_: Carbon dioxide
TVOC: Total volatile compounds
PM: Particulate matter
CO: Carbon monoxide
O_3_: Ozone
SO_2_: Sulphur dioxide
HCHO: Formaldehyde

## Additional Information and Declarations

### Data Availability

The following information was supplied regarding data availability: The raw data are available in Supplemental File.
